# Purification of pregnancy-associated glycoproteins from late-pregnancy *Bubalus bubalis* placentas and development of a radioimmunoassay for pregnancy diagnosis in water buffalo females

**DOI:** 10.1186/1746-6148-9-89

**Published:** 2013-05-01

**Authors:** Olimpia Barbato, Noelita Melo de Sousa, Vittoria Lucia Barile, Claudio Canali, Jean-François Beckers

**Affiliations:** 1 Department of Biopathological Veterinary Science, Faculty of Veterinary Medicine, University of Perugia, Perugia, 06126, Italy; 2 Laboratory of Animal Endocrinology and Reproduction, Faculty of Veterinary Medicine, University of Liege, Liege, 4000, Belgium; 3 Animal Production Research Centre, Italian Agricultural Research Council, Monterotondo, 00016, Italy

**Keywords:** Water buffalo, Placenta, Pregnancy-associated glycoprotein, Purification, Radioimmunoassay, Polyclonal antiserum, Pregnancy diagnosis, N-terminal amino acid sequence

## Abstract

**Background:**

Pregnancy-associated glycoproteins (PAGs) were first described as placental antigens present in the blood serum of the mother soon after implantation. Here, we describe the purification of several pregnancy-associated glycoproteins from water buffalo placenta (wbPAGs). A specific radioimmunoassay (RIA) was developed for early pregnancy diagnosis in buffalo species.

**Results:**

Amino-terminal microsequencing of immunoreactive placental proteins allowed the identification of eleven wbPAGs sequences [Swiss-Prot accession numbers: P86369 to P86379]. Three polyclonal antisera (AS#858, AS#859 and AS#860) were raised in rabbits against distinct wbPAG fractions. A new RIA (RIA-860) was developed and used to distinguish between pregnant (n = 33) and non-pregnant (n = 26) water buffalo females.

**Conclusions:**

Our results confirmed the multiplicity of PAG expression in buffalo placenta. In addition, the RIA-860 system was shown to be sensitive, linear, reproducible, accurate and specific in measuring PAG concentrations in buffalo plasma samples from Day 37 of gestation onwards.

## Background

Domestic buffalo are considered to have low reproductive efficiency, characterised by late attainment of puberty and maturity, seasonality of calving, long postpartum anoestrus, poor expression of oestrus signs, low conception rate and long calving intervals [1,2]. Furthermore, there is some evidence of a high rate of embryonic loss, in particular during the critical phase of embryonic attachment [3,4]. Thus, an accurate early distinction of pregnant and non-pregnant animals is essential for improvement of reproductive efficiency in buffalo, particularly when breeding techniques such as “out of breeding season mating” or artificial insemination (AI) are applied. Moreover, the study of embryonic mortality through the detection of pregnancy markers could support researchers aiming to improve oestrus synchronisation and fixed time AI programs in buffaloes.

Since the early 1980s, a large family of placental proteins without known biological activity (pregnancy-associated or pregnancy-specific proteins, namely pregnancy-associated glycoprotein (PAG) or pregnancy-specific protein B (PSPB)) has been purified from ruminant placenta [5,6]. Some of them are detected in the peripheral circulation of pregnant females, and are used as a tool to investigate placental function in ongoing or endangered pregnancies [7-9].

PAGs are expressed in mono- and binucleate trophoblastic cells of the outer epithelial layer in the synepitheliochorial cotyledonary placenta [10-13]. Using an antiserum (AS) raised against bovine PAG (AS#PAG-F4), Carvalho et al. [14] confirmed that there is a strong homology between water buffalo and ruminant binucleate cells concerning cell morphology, protein expression, glycosylation pattern and characteristics of cell migration and fusion. Buffalo binucleate cells migrate toward the maternal epithelium and fuse with a uterine epithelial cell to form a trinucleate cells [14]. Maternal hybrid trinucleate cells can also further fuse with adjacent cells, resulting in the formation of a multinuclear syncytium [15]. However, larger syncytia, with more than three nuclei, are much less frequent than trinucleate cells in buffalo placentas [14].

PAG molecules belong to the aspartic proteinase (AP) superfamily [16] and originated from an ancient PAG-like precursor by duplication and positive selection approximately 87 million years ago [17]. It was estimated that cattle, sheep, and probably other pecoran mammals possess many, possibly 100 or more, PAG genes [18]. To date, 74 different complementary DNA (cDNA) of PAG genes (differing by at least 5% in nucleotide sequence) have been identified in species with a synepitheliochorial placenta. In bovine species, 22 PAG genes (boPAG-1 to boPAG-22) have been cloned and fully sequenced [11,16,18,19]. The number of identified PAG polypeptide precursors is lower in ovine (11 ovPAG) [18,20], caprine (12 caPAG) [12,19], cervid (10 cePAG) [21] and water buffalo species (wtPAG-1 [22]; wtPAG-2 to wtPAG-19 [Green et al., GenBank direct submission]).

Molecular biology techniques have allowed for huge progress in understanding the phylogenetic diversity of PAG molecules. However, these techniques are not adequate to obtain purified and semi-purified PAG preparations necessary for the development of radioimmunoassay (RIA) and enzyme-linked immunosorbent assay (ELISA) protocols. Fortunately, since the 1990s, the selection of the most convenient chromatography procedures allowed for the isolation of an important number of native purified PAG. At present, forty PAG isoforms have been isolated from cotyledons of the cow [6,23,24], ewe [20,25,26], goat [27], buffalo [28], bison [29,30], moose and elk [31]. Some of them were used to immunise rabbits and the antisera obtained allowed the immunolocalisation of PAG in placental tissue [10,13,14,32] and the determination of secretory pattern measured in peripheral maternal blood (reviewed by Sousa et al. [33]).

In buffalo species, despite efforts made on the purification of PAG molecules, the quantity of purified PAG has not been sufficient to raise new PAG antisera. So far, PAG concentrations in buffalo species were determined by heterologous PAG-RIA systems based on antisera raised against bovine or caprine PAG [34,35]. Interestingly, by using these systems, concentrations of PAG were remarkably distinct from those measured in cattle, increasing gradually from the 6^th^ week of gestation to parturition (28^th^ week of pregnancy), and reaching relatively low peripartum levels [35].

Here, we describe the successful isolation and characterisation of new buffalo PAG molecules. These newly purified proteins were used for the production of three specific antisera to develop a new PAG-RIA system (RIA-860). Finally, its ability to discriminate between pregnant and non-pregnant females was evaluated in Italian Mediterranean buffalo cows.

## Results

### Isolation and characterisation of water buffalo PAG (wbPAG)

**Figure 1:**
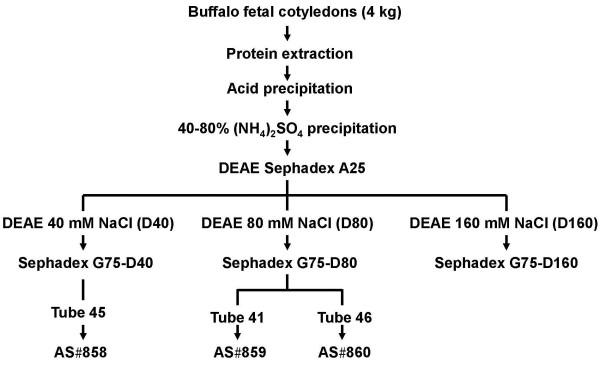
Schematic outline of PAG purification from the placentas removed at 8 months of gestation.

**Figure 2:**
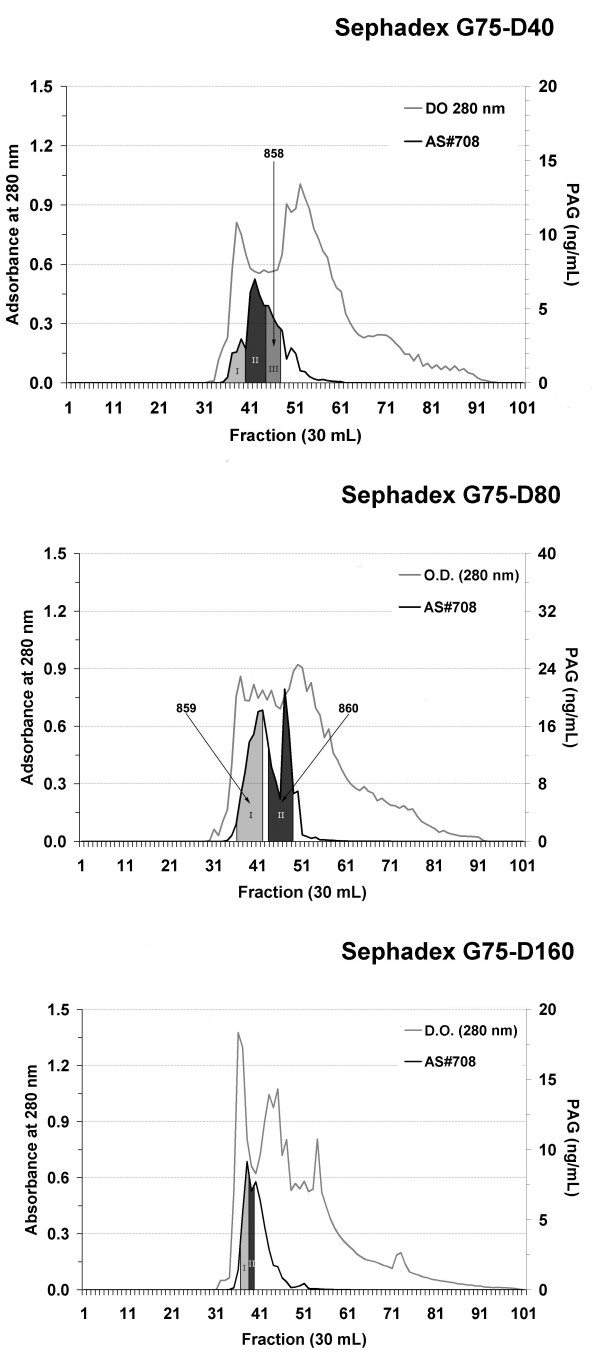
**Sephadex G-75 chromatographic profiles of 40, 80, and 160 mM NaCl-DEAE fractions from the buffalo placentas.** The column (5 × 100 cm) was previously equilibrated with 5 mM ammonium bicarbonate buffer (pH 7.5). Elution of proteins (O.D. at 280 nm) is indicated by a black line while immunoreactivity RIA-708) is indicated by a grey line. Stippled areas indicate the most immunoreactive fractions.

Figure 1 schematically shows the protocol used to isolate wbPAG from fetal cotyledons. Amounts of total protein (TP) and equivalent immunoreactive PAG contents (equivPAG; determined by RIA-708) in the different steps of wbPAG purification are summarised in Table 1. Highest immunoreactivity was observed at the DEAE 80 mM NaCl (D80) when compared to DEAE 40 mM NaCl (D40) and DEAE 160 mM NaCl (D160). Sephadex G75 peaks exhibiting the highest PAG/TP ratios (Figure 2) were loaded onto VVA lectin-affinity chromatography. The VVA chromatographies of Sephadex G75-D40 Peak III, G75-D80 Peak II and G75-D160 Peak I and II resulted in PAG/TP ratios higher than 100% (Table 2).

**Table 1:** Total protein, immunoreactive PAG and ratio of immunoreactive PAG/total protein in different steps of purification

**Purification steps**	**TP (mg)**	**equivPAG (mg)**	**PAG/TP ratio (%)**
Protein extraction	140,897.6	5,719.5	4.1
Acid precipitation	55,598.0	2,919.5	5.2
Ammonium sulfate 40–80%	19,828.5	3,025.5	15.3
DEAE 40 mM NaCl (D40)^a^	1,132.1	176.3	15.6
Sephadex G75-D40 peak I (tubes 36–39)	67.2	9.4	14.8
Sephadex G75-D40 peak II (tubes 40–43)	82.2	24.3	29.6
Sephadex G75-D40 peak III (tubes 44–47)	39.0	17.0	43.6
DEAE 80 mM NaCl (D80)^a^	2,481.3	824.2	33.2
Sephadex G75-D80 peak I (tubes 36–42)	146.4	95.4	65.1
Sephadex G75-D80 peak II (tubes 43–49)	129.8	74.7	57.5
DEAE 160 mM NaCl (D160)^a^	3,527.7	174.6	4.9
Sephadex G75-D160 peak I (tubes 37–38)	74.4	16.2	21.7
Sephadex G75-D160 peak II (tubes 39)	31.5	7.7	24.4

^a^Eight hundred milligrams from each DEAE were loaded on the Sephadex G75.

TP: total protein.

equivPAG: PAG concentrations measured by heterologous RIA.

**Table 2:** **Characteristics of different eluted fractions giving the highest ratio of PAG/TP obtained after chromatography on *Vicia villosa *agarose**

**Fraction used for VVA chromatography**	**PAG**^**a**^**/TP**^**b **^**ratio**	**MM**^**c **^**of major stained bands**		**N-terminal amino acid sequence**	**Code of protein**	**Accession number**
		**Coomassie staining**	**Western blot**			
D40-G75 peak III	> 100%	69	64	RGSXLTIHP	wbPAG_69kDa__A	P86372
D80-G75 peak I	65.8%	73	68	RGSXLTILPLRNKIDLFYVG	wbPAG_73kDa__B	P86373
		61	59	RGSXLTILPLRNIRDIFYVG	wbPAG_61kDa__C	P86374
D80-G75 peak II	> 100%	76	70	RGSXLTIHPL	wbPAG_76kDa__D	P86375
		65	60	RGSXLTH	wbPAG_65kDa__E	P86376
		58	55	RGSXLTHLP	wbPAG_58kDa__F	P86377
D160-G75 peak I	> 100%	76	70	RGSXLTIHP	wbPAG_76kDa__G	P86378
		63	60	RGSXLTIHP	wbPAG_63kDa__H	P86379
D160-G75 peak II	> 100%	73	68	RGSXLTHLPLRNISD	wbPAG_73kDa__I	P86371
		63	61	RGSXLTILPLRNISD	wbPAG_63kDa__J	P86370
		60	57	RGSXLTIHPL	wbPAG_60kDa__K	P86369

^a^Determined by radioimmunoassay. ^b^Obtained by weighing each fraction. ^c^Apparent molecular masses (MM) were estimated after SDS-PAGE and Western blot (AS#497 + AS#708, 0.1:0.1 ml, v/v).

**Figure 3:**
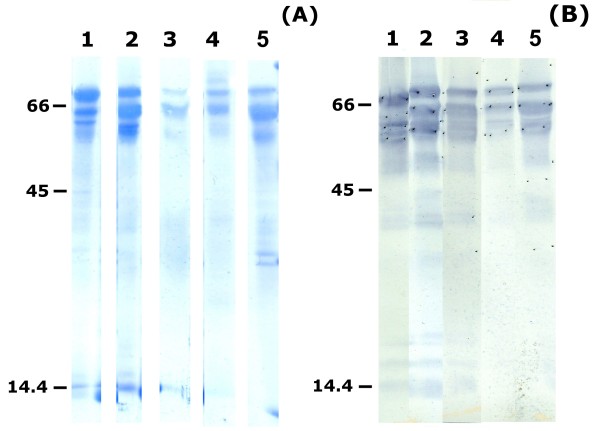
**Coomassie blue stained PVDF membrane after SDS-PAGE (A) and Western blot analysis with AS#708 (B).** Lane 1: VVA peak III from the 40 mM NaCl-DEAE fraction. Lane 2: VVA peak I from the 80 mM NaCl-DEAE fraction. Lane 3: VVA peak II from the 80 mM NaCl-DEAE fraction. Lane 4: VVA peak I from the 160 mM NaCl-DEAE fraction. Lane 5: VVA peak II from the 160 mM NaCl-DEAE fraction. Thirty (**A**) to 50 μg (**B**) were loaded in lanes 1–5. Molecular weight standards (kDa; 7 μg/lane) were loaded on the left and the right sides of each figure. Final dilution of PAG AS#708 was 1:2,000.

Table 2 summarises the different purification steps used to obtain the eleven N-terminal sequenced water buffalo PAG molecules (wbPAG), as well as their apparent molecular masses as determined after Coomassie staining of the PVDF membrane (Figure 3A) and Western blot (Figure 3B). The apparent molecular masses of immunoreactive PAG isoforms from VVA peaks (revealed by AS#708) ranged from 55 to 70 kDa. These molecular masses were systematically slightly lower than those revealed after Coomassie staining of the PVDF membrane (58 to 76 kDa). Proteins were submitted to Edman degradation based on their availability (Figure 3A).

The protein sequence data described are available in the UniProt knowledgebase under the accession numbers P86369 to P86379. Isolated wbPAG showed highly conserved amino acid residues at the beginning of the N-terminal extremity (RGS-), with Edman sequencing failing to give any signal on cycle 4 (Table 2). Buffalo sequenced N-termini contained the consensus PLR (residues 9 to 11).

When compared, the percentage of amino acid identity of newly sequenced wbPAG ranged from 80% (between wbPAG_73kDa__B and wbPAG_73kDa__I) to 100%. As shown in Table 2, in their sequenced part, wbPAG_65kDa__E, wbPAG_58kDa__F and wbPAG_73kDa__I were identical, as well as wbPAG_67kDa__A, wbPAG_76kDa__D, wbPAG_76kDa__G, wbPAG_63kDa__H and wbPAG_60kDa__K.

**Figure 4:**
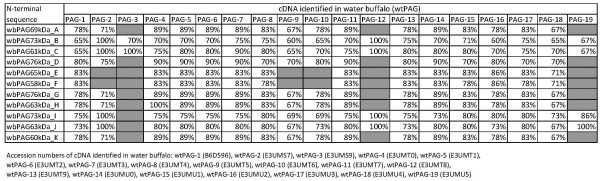
**Comparison of N-terminal amino acid sequences of newly isolated water buffalo PAG with sequences deduced from DNA databases.** Identities between two sequences are expressed as percent. Grey squares represent very low sequence identities.

**Figure 5:**
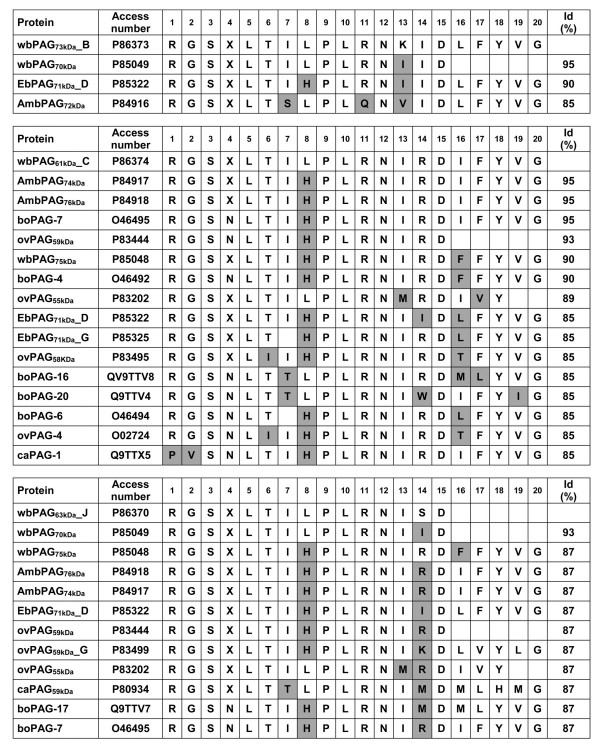
**Comparison of the N-terminal amino acid sequences of the newly isolated water buffalo PAG (wbPAG) with ruminant PAG exhibiting the highest sequence identity.** Peptide and DNA sequence databases were screened, and the identities (id) were determined by the EBI (European Bioinformatics Institute) using the FASTA3 network service. Grey square represent radical substitutions.

Screening of the EMBL and Swiss-Prot data banks revealed 100% identity between four PAG N-termini characterised here (wbPAG_73kDa__B, wbPAG_61kDa__C, wbPAG_73kDa__I and wb_63kDa__J) and those deduced from cDNA from buffalo species (Figure 4). Finally, the micro-sequence comparison also revealed that some wbPAG forms (wbPAG_73kDa__B, wbPAG_61kDa__C and wbPAG_63kDa__J) are unique compared to other purified proteins forms isolated from bovine, ovine, caprine and bison species (Figure 5).

### Development and validation of a new PAG RIA

**Figure 6:**
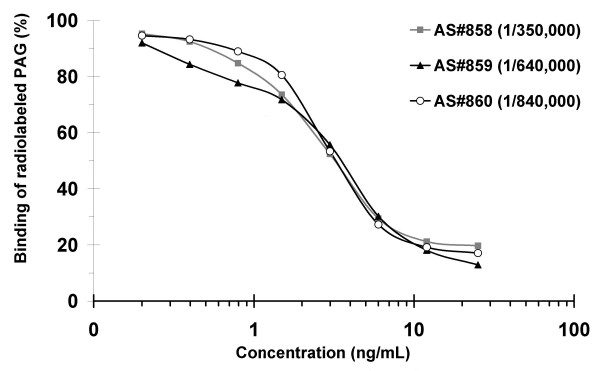
**Displacement of standard inhibition curves obtained with three different antisera raised against buffalo PAG**. A preparation of highly purified boPAG_67kDa_ was used as standard (0.2 to 25 ng/mL) and tracer (30,000 cpm/100 μL).

Figure 6 shows displacement of standard (0.2 to 25 ng/mL) inhibition curves (B/B_0_) tested using three different antisera raised against buffalo PAG (AS#858, AS#859 and AS#860). All tested antisera gave very similar slopes. Highest dilutions of primary antisera were obtained with AS#860 (1:840,000). Therefore, this system (RIA-860) was chosen for measuring concentrations of PAG in water buffalo cows.

**Figure 7:**
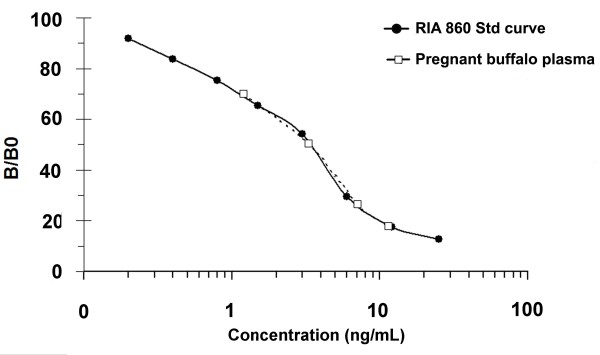
**Parallelism between standard curve and serial dilutions of pregnant buffalo plasma samples.** Standard curve for RIA-860 was calculated by linear scale of B/B_0_ ratio vs. decadic logarithm of the standard concentration using radiolabelled boPAG_67kDa_. Plasma samples from pregnant buffalo were serially diluted at 1/1, 1/2, 1/4, 1/8, and 1/16.

Concerning RIA-860 validation, MDL was 0.1 ng/mL. Parallelism between standard curve and serial dilutions from a pregnant buffalo female is demonstrated in Figure 7. Reproducibility measured as the coefficients of variation intra- and inter-assay was 6.7% (2.3 ± 0.2 ng/mL) and 8.0% (3.1 ± 0.2 ng/mL), respectively. Regarding specificity, the presence of different concentrations (0.19 until 1,000 ng/mL) of placental proteins, sugars and other plasmatic compounds did not alter the binding of radio-labelled PAG. Finally, regarding recovery, it ranged from 101 to 110% when concentrations of 10.0 and 2.0 ng/mL were added to a sample containing low PAG concentrations.

### Concentrations of PAG in plasma samples of pregnant and non-pregnant buffalo cows

From a total of 59 buffalo females used, 33 became pregnant after AI, as detected by both rectal palpation and RIA-860. As shown in Table 3, in non-pregnant animals, mean PAG concentrations remained under 0.5 ng/mL at Days 0, 30 and 37 after AI. Two non-pregnant females exhibited PAG concentrations of 1.0 ng/mL: one female at Day 0, and the other at Day 30 after AI. In the pregnant group, two females exhibited PAG concentrations under 1.0 ng/mL at Day 30 after AI. In this group, concentrations increased significantly from Day 0 to 30 and from Day 30 to 37, when the best threshold for discrimination between pregnant and non-pregnant animals was observed.

**Table 3:** Plasma concentrations of PAG measured by using AS#860 in non-pregnant and pregnant buffalo cows

**Day after AI**		**Concentrations of PAG (ng/mL)**	
		**Non-pregnant (n = 26)**	**Pregnant (n = 33)**
Day 0	Mean PAG (ng/mL)	0.48 ± 0.04	0.41 ± 0.04^a^
	[min–max]	[0-1.0]	[0-0.8]
Day 30	Mean PAG (ng/mL)	0.46 ± 0.06*	2.86 ± 0.39^a,b,*^
	[min–max]	[0-1.0]	[0.85-11.37]
Day 37	Mean PAG (ng/mL)	0.27 ± 0.05**	11.60 ± 1.61^b,**^
	[min–max]	[0-0.8]	[3.12-49.00]

PAG concentrations are expressed as mean ± SEM.

AI: artificial insemination.

Values with similar superscripts in the same column are statistically different (^a^P < 0.01; ^b^P < 0.05).

Significant differences between PAG concentrations from pregnant and non-pregnant females are indicated by asterisks (*P < 0.05; **P < 0.0001).

## Discussion

Purified native PAG preparations are required for the development of specific and/or more sensitive immunoassay techniques that are currently used for pregnancy diagnosis and physiopathological investigations in ruminant species [6,27,33]. Our work describes the isolation and characterisation of different PAGs from buffalo placentas. Some proteins were used for production of polyclonal antisera, allowing the development of new radioimmunoassay systems. In parallel, our work confirmed the large heterogeneity of PAG molecules for both molecular mass and N-terminal amino acid sequence.

Heterogeneity of the PAG subfamily was evoked as early as 1982, when Butler et al. [5] estimated molecular mass of bovine PSPB to range from 47 to 53 kDa. However, after several years, it was not clear if such diversity was due to the expression of distinct PSPB/PAG forms in ruminant placenta, to an extensive post-translational processing in placental tissue (glycosylation, phosphorylation or others), or to both phenomena. The identification of a high number of N-terminal sequences and cDNA corresponding to different ruminant PAG and the demonstration of an extensive glycosylation mechanism in bovine and ovine placenta [36-38] confirmed that both factors contribute to the PAG diversity described here and in previous works [24-30]. For instance, different numbers of N-glycosylation sites of asparagines have been observed in PAG isoforms identified in placentas of cattle, sheep and goats [6,11,12,32,37]. In cattle, the dominant boPAG_67kDa_ form has been shown to present 4 potential glycosylation sites and 10% of oligosaccharides content [6]. Multi-antennary oligosaccharides have been shown to represent 17.83% of the relative molecular mass of ovPAG [37]. The number of potential sites of glycosylation of buffalo PAG had not yet been described in the literature. However, as previously reported, N-terminal sequences of wbPAG failed to give any signal on cycle 4, this blank cycle being followed by the consensus sequence L-T that is a characteristic of the N-glycosylation site.

Our results confirm earlier findings of Klisch et al. [24], Barbato et al. [28] and Kiewiesz et al. [29,30], who reported that VVA chromatography can be very useful to enrich placental glycoproteins produced by binucleate cells. Interestingly, despite several PAG sequences have been obtained in buffalo species, none of them corresponded to the cDNA sequence of buffalo PAG-1 (SwissProt access number B6D596). In bovine species (*Bos taurus taurus* and *Bos taurus indicus*), when using the classical purification protocol, a single major protein (PAG-1) was identified in placental extracts [6,23]. On the other side, a large heterogeneity of PAG molecules could be identified in placental extracts from small ruminants, European and American bison [25-27,29,30].

Concerning analysis of N-terminal amino acid sequences of newly purified buffalo PAGs, the interpretation was limited by the relatively low number of residues clearly identified (7 to 20 amino acids long). Three of them (wbPAG_65kDa__E, wbPAG_58kDa__F and wbPAG_73kDa__I) were identical to wbPAG_62kDa,_ previously isolated from mid-pregnancy (5 to 6 months) buffalo placenta [28]. Five other sequences (wbPAG_67kDa__A, wbPAG_76kDa__D, wbPAG_76kDa__G, wbPAG_63kDa__H and wbPAG_60kDa__K) were 100% identical to proteins previously characterised in buffalo (wbPAG_73kDa_ and wbPAG_75kDa_) [28], ovine (ovPAG59, -58a, -61b, 60f, -59g) [25,26] and bison (AmbPAG_74kDa_, AmbPAG_76kDa_, EbPAG_71kDa__D) placentas [29,30].

Our work describes for the first time the production of polyclonal antisera raised against PAG molecules isolated from buffalo placenta. Proteins issued from three different fractions (DEAE 40 mM NaCl, Sephadex G75-Peak III; DEAE 80 mM NaCl, Sephadex G75-Peak I and DEAE 80 mM NaCl, Sephadex G75-Peak II) were used to immunise rabbits resulting in the production of three distinct antibodies: AS#858, AS#859 and AS#860, respectively. These three obtained antisera gave very similar displacement of standard inhibition curves. This finding was not surprising because PAG molecules expressed in the same species can exhibit high sequence identities [20,25,26,30], and thus probably share common epitopes [11,33].

The highest dilution of primary antiserum (1:840,000) was obtained with AS#860. The remarkable parallelism between serial dilutions of pregnant buffalo samples and the standard curve indicates that RIA-860 is a good immunoassay allowing distinguishing subtle quantitative differences in wbPAG concentrations. RIA-860 also proved to be very repeatable for measurement of PAG concentrations (intra- and inter-assay CV lower than 8%). Finally, it was observed that MDL of RIA-860 was equivalent (0.1 ng/mL) to that previously described for RIA-497 [39], RIA-706 [39-41] and RIA-Pool [41].

The use of PAG/PSPB RIA systems are considered as reliable method for early pregnancy diagnosis and follow-up of trophoblastic function in ruminant species (reviewed by Sousa et al. [33]). Measurements of PAG/PSPB concentrations in the peripheral circulation of pregnant and non-pregnant buffalo cows have been performed using different RIA methods [35,42]. Recently, Barbato et al. [35] compared RIA-497, RIA-706 and RIA-708 (antisera raised against boPAG_67kDa_, caPAG_55+59kDa_ and caPAG_55+62kDa_, respectively) for detecting PAG molecules in pregnant buffalo females. They reported that analogous PAG antigens were better recognised using RIA-706 from week 6 of gestation onwards. By using the same PAG-RIA system, Karen et al. [34] described that pregnancy diagnosis reached 100% between days 31 and 35.

An additional feature of the present study was the observation of a rapid increase in PAG concentrations from Day 30 (2.9 ± 0.4 ng/mL) to Day 37 of pregnancy (11.6 ± 1.6 ng/mL). Interestingly, a rapid increase in PAG concentration during pregnancy is characteristic of ovine [43] and caprine species [44]. However, it differs largely from those described in bovine species, in which PAG concentrations increase slowly during the first trimester of gestation [39,45].

In Egyptian buffalo cows, Karen et al. [34] and El-Battawy et al. [42] described mean PAG concentrations ranging from 6.4 to 9.8 ng/mL (15 and 5 pregnant females, respectively) at around Day 30 of pregnancy. Those values were higher than mean concentrations (2.9 ng/mL) measured by RIA-860 at Day 30 in Italian Mediterranean females (n = 33). Egyptian and Mediterranean buffalo cows are quite different in several aspects. Mediterranean females have a much higher milk yield than Egyptian females (12 vs. 5 kg/day) [46,47]. This observation of low PAG levels in high producing milk females agrees with previous findings described by Lopez-Gatius et al. [48] who reported that there is a strong negative correlation between milk production and PAG concentrations during the first trimester of pregnancy. Differences in PAG concentrations could also be partially due to other factors such as breed, placental mass, age of female or even farm (genetic selection and nutritional management) [49]. Moreover, as previously described in bovine and ovine species, PAG expression is complex throughout pregnancy. Some PAG molecules are expressed early, while others only as pregnancy progresses. Other PAG are also expressed throughout the whole pregnancy period [11].

PAG concentrations were detectable in peripheral plasma of all Italian Mediterranean pregnant buffalo cows at Day 37. At Day 30, 31 out of 33 pregnant females were already positive by RIA-860. In dairy and beef cows, PAG concentrations can be detected as early as at Day 21 after AI [39]. However, pregnancy diagnosis is only recommended from Day 28 to 30 because of important individual variation on PAG appearance in peripheral blood [50]. According to different authors, expression of PAG family members as early as Day 7 after fertilisation suggests their potential role in cellular growth and differentiation, elongation, apposition, attachment and placentogenesis processes [51-54].

On the other hand, in the present study, PAG concentrations were higher than 1.0 ng/mL in one non-pregnant female at Days 0 and 30. Recently, Karen et al. [34] described pregnancy diagnosis in Egyptian buffalo cows by using RIA-706 (antiserum raised against caPAG_55+62kDa_). The authors also reported 3/39 and 2/35 incorrect pregnancy diagnoses between days 31–35 and 36–40, respectively. The presence of detectable concentrations in non-pregnant females can be explained by the existence of extra-placental sources of PAG, as suggested by Zoli et al. [50]. Indeed, the presence of antigens immunologically related to PAG has been demonstrated in testicular and in ovarian extracts, justifying, also, the adjective “associated” and not “specific” given to this family of placental glycoproteins [55].

## Conclusion

We have described for the first time the use of antisera raised against buffalo PAG for RIA development and pregnancy detection in buffalo cows. Moreover, we showed that RIA-860 was quantitative, precise, accurate and sensitive in measuring PAG concentrations.

## Methods

### Purification of pregnancy-associated glycoproteins from buffalo placenta

Throughout the procedure, the presence of immunoreactive proteins was screened by PAG RIA-708 and Western blot techniques, as previously described [28]. Total protein (TP) concentrations were determined by the Lowry method [56] with bovine serum albumin (BSA; ICN Biochemicals Inc., Aurora, OH, USA) as the standard. After Sephadex G-75 and VVA chromatographies, protein contents were monitored by measuring the UV absorption at 280 nm.

Two placentas were collected from pregnant buffalo cows (*Bubalus bubalis*) immediately after slaughter, washed with 0.9% NaCl and frozen at –20°C. The stage of pregnancy was 8 months (determination based on the day of artificial insemination). Approximately 4 kg of fetal cotyledons were thawed, finely minced, mixed and homogenised (2 h, 4°C) in 10 mM potassium phosphate buffer containing 100 mM KCl (pH 7.6; ratio buffer to tissue 4:1, v:v) in the presence of protease inhibitors (PMSF, EDTA, NaN_3_). After centrifugation (16,000 × g, 1 h, 4°C), a second extraction followed by homogenisation (2 h, 4°C) was performed. The pellet obtained after centrifugation was thawed and frozen five times, before a third extraction in phosphate buffer.

Supernatants issued from the three extractions were pooled, adjusted to pH 4.5 with 0.5 M H_3_PO_4_ and allowed to precipitate at 4°C overnight. After centrifugation, the pH of the supernatant was readjusted to 7.6 with 0.5 M KOH solution. Extracted proteins were then precipitated by ammonium sulphate ((NH_4_)_2_SO_4_) at 40% saturation (16 h, 4°C) and centrifuged. Additional ammonium sulfate was added to achieve 80% of saturation (40–80% (NH_4_)_2_SO_4_ fraction). Following 4 h precipitation, the solution was centrifuged (16,000 × g) and the pellet was suspended in 10 mM Tris-HCl buffer (pH 7.6), extensively dialysed against the same buffer (48 h) and centrifuged (16,000 × g, 1 h) to eliminate the insoluble proteins.

The 40–80% (NH_4_)_2_SO_4_ precipitate was loaded onto a chromatographic column (14 × 25 cm, 3,800 mL) of DEAE-Sephadex A25 (Amersham Biosciences, Uppsala, Sweden) previously equilibrated on 10 mM Tris-HCl buffer (pH 7.6). Five steps of increasing ionic-strength buffer (20, 40, 80, 160 and 320 mM NaCl) were used for the elution of the column. After chromatography, each fraction was concentrated to a final volume of 200 mL, extensively dialysed against 5 mM ammonium bicarbonate buffer (pH 8) and lyophilised. Therefore, isolation was followed independently for the DEAE 40 mM NaCl (D40), DEAE 80 mM NaCl (D80) and DEAE 160 mM NaCl fractions (D160).

Batches of 800 mg of proteins issued from D40, D80 and D160 were subjected to gel filtration on a Sephadex G75 column (5 × 100 cm; Amersham Biosciences) equilibrated in 5 mM ammonium bicarbonate buffer. After each gel filtration, proteins from the tube presenting the highest immunoreactivity were lyophilised and used to immunise rabbits (Figure 2). The other tubes corresponding to rich fractions were pooled together and dialysed against 10 mM HEPES buffer (pH 7.5). After dialysis, each rich fraction was run through an 8 mL agarose-bound *Vicia villosa* lectin (VVA) column (0.7 × 20 cm; Vector Laboratories, Burlingame, CA, USA). Proteins were eluted with 80 mL HEPES buffer containing 50 mM N-acetyl-galactosamine (GalNAc; Acros Organics, Morris Plains, NJ, USA). All VVA eluted fractions were pooled, dialysed against 5 mM ammonium bicarbonate (pH 8), centrifuged (27,000 × g, 15 min) and lyophilised.

The VVA-eluted proteins were separated by one-dimensional SDS-PAGE. They were either visualised by Coomassie Brilliant Blue R250 staining, transferred to PVDF membranes for NH_2_-terminal microsequencing or transferred to nitrocellulose membranes for Western blot analysis as described elsewhere [25]. Amino acid (aa) micro-sequencing analyses were performed by Edman degradation on a pulsed liquid-phase protein sequencer (Procise 492; Applied Biosystems Inc., Foster City, CA, USA). The N-terminal sequences obtained in water buffalo placentas were deposited in the SwissProt database (access numbers P86369 to P86379). The NH_2_-terminal aa sequences of isolated PAG were compared with previously deposited full-length sequences of polypeptide PAG precursors identified from cloned cDNAs (GenBank) and to the micro-sequences of identified native PAG forms (EMBL-EBI). The comparison between N-terminal amino acid microsequence and those deduced from cDNA was performed using Blast program from NCBI. Identities were determined by the EBI (European Bioinformatics Institute) using the Fasta 3 network service [57]. Since it is known that the X in position 4 is part of a N-glycosylation site in PAG, it was substituted by asparagines (N) for database searches [18].

### Antisera production and determination of their dilutions for use in routine RIA

Three mature New Zealand white rabbits (AS#858, AS#859 and AS#860) were immunised with distinct purified PAG preparations (Figure 1) by intradermal route [58]. For the first immunisation, 300 μg of proteins were dissolved in 1.0 mL phosphate buffer 0.5 M (pH 7.5) and emulsified with Freund complete adjuvant (Difco Labs, Detroit, MI, USA). Booster doses (300 μg) were injected at 3–4 week intervals (Freund incomplete adjuvant). Blood was collected from the marginal ear vein starting one month after the second injection and then once a month. Rabbit blood samples were allowed to clot overnight at room temperature. Thereafter, they were centrifuged at 1,500 × *g* for 20 min, and the sera were stored at –20°C until used. The immunisation protocol was approved by the Animal Ethics Committee of the University of Liege (Dossier number 95).

In the presence of an excess of antibody, 44% (AS#858), 45% (AS#859) and 40% (AS#860) of labelled bovine 67 kDa PAG (boPAG_67kDa_) were bound. These antisera were tested at different dilutions to obtain a tracer-binding ratio in the zero standard (B_0_) of approximately 20% (B_0_/Tc) and a low non-specific binding (NSB < 1%). The optimal binding ratios were obtained at initial dilutions of 1/350,000 (AS#858), 1/640,000 (AS#859) and 1/840,000 (AS#860). The antiserum giving the highest dilution titre (AS#860) was used for PAG-RIA development and measurement in plasma samples from buffalo cows.

### PAG radioimmunoassay procedure

The PAG measurements were performed according to the method described by Zoli et al. [50] with some modifications. All assays were performed in Tris-HCl buffer (adjusted to pH 7.6) containing 0.1% BSA (Fraction V; Sigma-Aldrich Co., St Louis, MO, USA). Measurements were performed in duplicate in polystyrene tubes and incubations were performed at room temperature (20 to 22°C). Bovine PAG 67 kDa preparation (boPAG_67kDa_, accession number Q29432) was used as standard and tracer for all assays. Pure stock boPAG_67kDa_ (lyophilised powder) was diluted with assay buffer to give standard curves ranging from 0.2 to 25 ng/mL (preincubated system). Iodination (Na-I^125^, Amersham Biosciences) was carried out according to the Chloramine T method [59]. The double antibody precipitation system was composed of a mixture of sheep anti-rabbit immunoglobulin (0.83% v:v), normal rabbit serum (0.17% v:v), polyethylene glycol 6000 (20 mg/mL; Fluka Biochemika, Buchs, Switzerland), cellulose microcrystalline (0.05 mg/mL; Merck, Darmstad, Germany) and BSA (2 mg/mL) diluted in Tris buffer (25 mM Tris, 10 mM MgCl_2_ and 0.02% w/v NaN_3_; pH 7.5).

Briefly, standard and plasma samples (0.1 mL) were diluted in 0.1 mL and 0.2 mL of Tris-BSA buffer, respectively. Virgin buffalo heifer serum (PAG-free serum; 0.1 mL) was added to each tube of the standard curve. The maximum binding (B_0_) was determined by replacing standard preparations by 0.1 mL of assay buffer. The NSB tubes contained 0.3 mL of buffer and 0.1 mL of PAG-free serum. After the addition of an appropriate dilution of antiserum (AS#860 at 1:840,000; 0.1 mL), the serum samples and the standard tubes were incubated overnight at room temperature (20 to 22°C). The following day, 0.1 mL of I^125^-PAG (≈ 25,000 cpm) was added and the tubes were incubated for 4 h at room temperature. For separation of bound and free fractions, 1 mL of second antibody polyethylene glycol (PEG) solution was added to all the tubes and a further incubation (30 min) at room temperature was performed. The tubes were then washed with 2 mL of assay buffer and centrifuged at 2,500 × g at 4°C for 30 min. The supernatant was discarded and the pellet was washed again and counted in a gammacounter (Packard Cobra II AutoGamma, Milan, Italy). The same person handled the entire experimental protocol. The results were expressed as the ratio (%) between the amounts of tracer bound to antibody in the presence (B) and in the absence (B_0_) of unlabelled PAG.

### Validation of PAG radioimmunoassay

The minimum detection limit (MDL) was defined as the minimum amount of unlabelled PAG that caused a reduction in the percentage of tracer bound to the antibody greater than twice the standard deviation of 20 determinations of B_0_.

Parallelism was assessed by serially diluting pregnant buffalo serum containing relatively high PAG concentrations with PAG-free serum. Parallelism for each RIA system was determined by evaluating a sample at its initial strength (1/1), and at dilutions of 1/2, 1/4, 1/8 and 1/16.

Reproducibility was determined by calculating the intra- and inter-assay coefficients of variation (CV) as follow: [%CV = (SD/mean)*100]. For intra-assay CV, the same serum was assayed 10 times within the same assay. The inter-assay reproducibility was assessed by analysing each serum in four consecutive assays.

Accuracy was determined by adding increasing concentrations of purified boPAG_67_ (1.0, 2.0, 4.0 and 10.0 ng) to buffalo sera containing known PAG concentrations. These amounts were chosen to be in the range of PAG concentrations generally found during early pregnancy. The percentage of recovery was calculated as follows: [observed value (ng/mL) / expected value (ng/mL)] × 100.

The specificity was verified by testing proteins such as bovine and sheep haemoglobin (Catalogue number H 2500 and H 2750; Sigma-Aldrich Co.) and serum albumins from bovine species (Fraction V; Catalogue number A4503, Sigma-Aldrich Co.), as well as the following carbohydrate preparations: N-acetyl-D-galactosamine (Ref. 22585; Acros Inorganics, Geel, Belgium), N-acetyl-D-glucosamine (Ref. A8625; Sigma-Aldrich Co.) and N-acetylneuraminic acid (from sheep sub maxillary gland ≥ 99%; Ref. A 9646; Sigma-Aldrich Co.).

### Collection of buffalo plasma samples and experimental design

The trial was carried out at the experimental farm of the Animal Breeding Research Center of Monterotondo (Rome, Italy, 42° N parallel). Fifty-nine Italian Mediterranean buffalo cows of different ages and parity were used for determination of PAG concentrations. The animals were housed in an open paddock, fed *ad libitum* on total mixed ration based on maize silage, alfalfa hay, soya bean meal, maize meal and barley meal (containing 0.90 UFL/Kg of dry matter (DM) and 15% crude protein on DM) and milked twice daily.

The buffalo cows were treated with a progesterone-releasing intravaginal device (PRID®; Sanofi, France), containing 1.55 g natural progesterone and a gelatine capsule with 10 mg oestradiol benzoate (which was included within the device). PRID® was kept in place for 10 days. On the 7^th^ day after PRID® insertion, an i.m. injection of 1000 IU of PMSG (Ciclogonina®, Fort Dodge, Italy) and 0.15 mg of cloprostenol (PGF2alpha analogue) (Dalmazin®, FATRO, Italy) were given. Buffaloes were artificially inseminated using frozen-thawed semen at 72 and 96 h after PRID® removal. Pregnancy status was confirmed by rectal palpation 40 days after AI.

Blood was withdrawn at Days 0, 30 and 37 after artificial insemination (AI). Approximately 10 mL were collected from the jugular vein into EDTA coated tubes. Plasma was separated by centrifugation at 2,500 × g for 10 min, and stored at –20°C until assayed. Blood sampling was performed in accordance with good veterinary practices and approved by the Animal Ethics Committee of the University of Perugia.

Statistical analysis was carried out by using Student t-test. Concentrations of PAG in pregnant and non-pregnant buffalo females were expressed as the means ± standard error of mean (SEM). Statistical significance was considered at the P < 0.05 level.

## Competing interests

The authors declare that they have no competing interests.

## Authors’ contributions

OB performed experimental work, data analysis and drafted the manuscript. NMS participated in carrying out PAG purification and sequence analysis, and had important input into and participation in writing the manuscript. VLB contributed to data collection and commented on the manuscript. CC assisted in the design of study and participated in carrying out radioimmunoassays. JFB conceived the design of the study, coordinated the work and helped in writing the manuscript. All authors read and approved the final version of the manuscript.

## References

[B1] Barile VL Borghese A Reproductive efficiency in female buffaloes Buffalo Production and Research. FAO Technical Series 2005 77 108

[B2] Perera BMAO Reproductive cycles of buffalo Anim Reprod Sci 2011 124 194 199 10.1016/j.anireprosci.2010.08.022 20869822

[B3] Campanile G Neglia G Gasparrini B Galiero G Prandi A Di Palo R D’Occhio MJ Zicarelli L Embryonic mortality in buffaloes synchronized and mated by AI during the seasonal decline in reproductive function Theriogenology 2005 63 2334 2340 10.1016/j.theriogenology.2004.10.012 15826694

[B4] Campanile G Del Vecchio D Di Palo R Neglia G Gasparrini G Prandi A Zicarelli L D’Occhio MJ Delayed treatment with GnRH agonist, hCG and progesterone and reduced embryonic mortality in buffaloes Theriogenology 2008 70 1544 1549 10.1016/j.theriogenology.2008.07.003 18706685

[B5] Butler JE Hamilton WC Sasser RG Ruder CA Hass GM Williams RJ Detection and partial characterization of two bovine pregnancy-specific protein Biol Reprod 1982 26 925 933 10.1095/biolreprod26.5.925 6807365

[B6] Zoli AP Beckers JF Wouters-Ballman P Closset J Falmagne P Ectors F Purification and characterization of a bovine pregnancy-associated glycoprotein Biol Reprod 1991 45 1 10 10.1095/biolreprod45.1.1 1908709

[B7] Serrano B López-Gatius F Hunter RH Santolaria P García-Ispierto I Bech-Sabat G de Sousa NM Beckers JF Yániz JL Anomalous pregnancies during late embryonic/early foetal period in high producing dairy cows Reprod Domest Anim 2009 44 672 676 10.1111/j.1439-0531.2007.01045.x 18694426

[B8] García-Ispierto I Nogareda C Yániz JL Almería S Martínez-Bello D de Sousa NM Beckers JF López-Gatius F *Neospora caninum* and *Coxiella burnetii* seropositivity are related to endocrine pattern changes during gestation in lactating dairy cows Theriogenology 2010 74 212 220 10.1016/j.theriogenology.2010.02.004 20416940

[B9] Breukelman SP Perényi Z Taverne MA Jonker H van der Weijden GC Vos PL de Ruigh L Dieleman SJ Beckers JF Szenci O Characterisation of pregnancy losses after embryo transfer by measuring plasma progesterone and bovine pregnancy-associated glycoprotein-1 concentrations Vet J 2012 194 71 76 10.1016/j.tvjl.2012.02.020 22516919

[B10] Zoli AP Demez P Beckers JF Reznik M Beckers A Light and electro microscopic immunolocalization of bovine pregnancy associated glycoprotein in the bovine placentome Biol Reprod 1992 46 623 629 10.1095/biolreprod46.4.623 1576259

[B11] Green JA Xie S Quan X Bao B Gan X Mathialogan N Beckers JF Roberts RM Pregnancy-associated bovine and ovine glycoproteins exhibit spatially and temporally distinct expression patterns during pregnancy Biol Reprod 2000 62 1624 1631 10.1095/biolreprod62.6.1624 10819764

[B12] Garbayo JM Green JA Manikklam M Beckers JF Kiesling DO Ealy AD Roberts RM Caprine pregnancy-associated glycoprotein (PAG): their cloning, expression and evolutionary relationship to other PAG Mol Reprod Dev 2000 57 311 322 10.1002/1098-2795(200012)57:4<311::AID-MRD2>3.0.CO;2-F 11066059

[B13] Wooding FB Roberts RM Green JA Light and electron microscope immunocytochemical studies of the distribution of pregnancy associated glycoproteins (PAGs) Placenta 2005 26 807 827 10.1016/j.placenta.2004.10.014 16226131

[B14] Carvalho AF Klisch K Miglino MA Pereira FT Bevilacqua E Binucleate trophoblast giant cells in the water buffalo (*Bubalus bubalis*) placenta J Morphol 2006 267 50 56 10.1002/jmor.10387 16240388

[B15] Takahashi T Hayashi K Hosoe M Biology of the placental proteins in domestic ruminants: expression, proposed roles and practical applications Japan Agric Res Quat 2013 47 43 45

[B16] Xie S Low BG Nagel RJ Kramer KK Anthony RV Zoli AP Beckers JF Roberts RM Identification of the major pregnancy-specific antigens of cattle and sheep as inactive members of the aspartic proteinase family Proc Natl Acad Sci USA 1991 88 10247 10251 10.1073/pnas.88.22.10247 1946444 PMC52905

[B17] Hughes AL Green JA Garbayo JM Roberts RM Adaptive diversification within a large family of recently duplicated, placentally expressed genes Proc Natl Acad Sci USA 2000 97 3319 3323 10.1073/pnas.97.7.3319 10725351 PMC16237

[B18] Xie S Green JA Bixby JB Szafranska B DeMartini JC Hecht S Roberts RM The diversity and evolutionary relationship of the pregnancy-associated glycoproteins, an aspartic proteinase subfamily consisting of many trophoblast-expressed genes Proc Acad Sci USA 1997 94 12809 12816 10.1073/pnas.94.24.12809 PMC24220 9371757

[B19] Garbayo JM Serrano B Lopez-Gatius F Identification of novel pregnancy-associated glycoproteins (PAG) expressed by the perimplantion conceptus of domestic ruminants Anim Reprod Sci 2008 103 120 134 10.1016/j.anireprosci.2006.12.002 17204380

[B20] Xie S Green JA Bao B Beckers JF Valdez KE Hakami L Roberts RM Multiple pregnancy-associated glycoproteins are secreted by day 100 ovine placental tissue Biol Reprod 1997 57 1384 1393 10.1095/biolreprod57.6.1384 9408244

[B21] Brandt GA Parks TE Killian G Ealy AD Green JA A cloning and expression analysis of pregnancy-associated glycoproteins expressed in trophoblasts of the white-tail deer placenta Mol Reprod Dev 2007 74 1355 1362 10.1002/mrd.20669 17393426

[B22] Jerome A Singh SK Agarwal SK Mohini S Raut A Characterization and *In Silico* analysis of pregnancy-associated glycoprotein-1 gene of buffalo (*Bubalus bubalis*) Genet Res Int 2011 2011 436138 (7 pages) 22567354 10.4061/2011/436138 PMC3335542

[B23] Sousa NM Remy B El Amiri B De Figueiredo JR Banga-Mboko H Goncalves PBD Beckers JF Characterization of pregnancy-associated glycoproteins extracted from zebu (*Bos indicus*) placentas removed at different gestational period Reprod Nutr Dev 2002 42 227 241 10.1051/rnd:2002021 12405451

[B24] Klisch K Sousa NM Beckers JF Leiser R Pich A Pregnancy-associated glycoprotein -1, -6, -7 and -17 are major products of bovine binucleate trophoblast giant cells at midpregnancy Mol Reprod Dev 2005 71 453 460 10.1002/mrd.20296 15822115

[B25] El Amiri B Remy B Sousa NM Joris B Otthiers NG Perenyi Z Banga Mboko H Beckers JF Isolation and partial characterization of three pregnancy-associated glycoproteins from ewe placenta Mol Reprod Dev 2003 64 199 206 10.1002/mrd.10246 12506352

[B26] El Amiri B Remy B De Sousa NM Beckers JF Isolation and characterization of eight pregnancy-associated glycoproteins present at high levels in the ovine placenta between day 60 and day 100 of gestation Reprod Nutr Dev 2004 44 169 181 10.1051/rnd:2004025 15460157

[B27] Garbayo JM Remy B Alabart JL Folch J Wattiez R Falmagne P Beckers JF Isolation and partial characterization of a pregnancy-associated glycoprotein family from the goats placenta Biol Reprod 1998 58 109 115 10.1095/biolreprod58.1.109 9472930

[B28] Barbato O Sousa NM Klisch K Clerget E Debenedetti A Barile VL Malfatti A Beckers JF Isolation of new pregnancy-associated glycoproteins from water buffalo (*Bubalus bubalis*) placenta by *Vicia villosa* affinity chromatography Res Vet Sci 2008 85 457 466 10.1016/j.rvsc.2008.01.004 18308351

[B29] Kiewisz K Sousa NM Beckers JF Vervaecke H Panasiewics G Szafranska B Isolation of pregnancy-associated glycoproteins from placenta of the American bison (*Bison bison*) Gen Comp Endocrin 2008 155 164 175 10.1016/j.ygcen.2007.04.011 17543308

[B30] Kiewisz K Sousa NM Beckers JF Panasiewics G Gizejewskiz Szafranska B Identification of multiple pregnancy-associated glycoproteins (PAGs) purified from European bison (Eb; Bison bison) placenta Anim Reprod Sci 2009 112 229 250 10.1016/j.anireprosci.2008.04.021 18538513

[B31] Huang F Cockrell DC Stephenson TR Noyes JH Sasser RG Isolation, purification and characterization of pregnancy-associated glycoprotein B from elk and moose placenta Biol Reprod 1999 61 1056 1061 10.1095/biolreprod61.4.1056 10491644

[B32] Klisch K Leiser R In bovine binucleate giant cells, pregnancy-associated glycoproteins and placenta prolactin-related protein-I are conjugated to asparagine-linked N-acetylgalactosaminyl glycans Histochem Cell Biol 2003 119 211 217 12649735 10.1007/s00418-003-0507-6

[B33] Sousa NM Ayad A Beckers JF Gajewski Z Pregnancy-associated glycoproteins (PAG) as pregnancy markers in the ruminants J Physiol Pharmacol 2006 57 Suppl 8 153 171 17242480

[B34] Karen A Darwish S Ramoun A Tawfeek K Van Han N Sousa NM Sulon J Szenci O Beckers JF Accuracy of ultrasonography and pregnancy-associated glycoprotein test for pregnancy diagnosis in buffaloes Theriogenology 2007 68 1150 1155 10.1016/j.theriogenology.2007.08.011 17884156

[B35] Barbato O Sousa NM Malfati A Debenedetti A Todini L Barile VL Beckers JF O’Leary M, Arnett J Concentrations of pregnancy-associated glycoproteins in Water buffaloes females (*Bubalus bubalis*) during pregnancy and postpartum periods Pregnancy Protein Research 2009 Washington DC: Nova Science Publishers 123 134

[B36] Gogolin-Ewens KJ Lee CS Mercer WR Moseby AM Brandon MR Characterization of a sheep trophoblast-derived antigen first appearing at implantation Placenta 1986 7 243 255 10.1016/S0143-4004(86)80162-X 3526314

[B37] Atkinson YH Gogolin-Ewens KJ Hounsell EF Davies MJ Brandon MR Seamark RF Characterization of placentation-specific binucleate cell glycoproteins possessing a novel carbohydrate. Evidence for a new family of pregnancy-associated molecules J Biol Chem 1993 268 26679 26685 8253801

[B38] Klisch K Wooding FB Jones CJ The glycosylation pattern of secretory granules in binucleate trophoblast cells is highly conserved in ruminants Placenta 2010 31 11 17 10.1016/j.placenta.2009.11.001 19959226

[B39] Perenyi Z Szenci O Sulon J Drion PV Beckers JF Comparison of the ability of three radioimmunoassay to detect pregnancy-associated glycoproteins in bovine plasma Reprod Domest Anim 2002 37 100 104 10.1046/j.1439-0531.2002.00341.x 11975748

[B40] Perenyi Z Szenci O Drion PV Banga-Mboko H Sousa NM El Amiri B Beckers JF Aspartic proteinase members secreted by the ruminant placenta: specificity of three radioimmunoassay systems for the measurement of pregnancy-associated glycoproteins Reprod Domest Anim 2002 37 324 329 10.1046/j.1439-0531.2002.t01-1-00366.x 12464069

[B41] Ayad A Sousa NM Sulon J Iguer-Ouada M Beckers JF Comparison of five radioimmunoassay systems for PAG measurement: ability to detect early pregnancy in cows Reprod Domest Anim 2007 42 433 440 10.1111/j.1439-0531.2006.00804.x 17635783

[B42] El-Battawy KA Sousa NM Szenci O Beckers JF Pregnancy-associated glycoprotein profile during the first trimester of pregnancy in Egyptian Buffalo cows Reprod Domest Anim 2009 44 161 166 10.1111/j.1439-0531.2007.00941.x 19192213

[B43] Ledezma-Torres RA Beckers JF Holtz W Assessment of plasma profile of pregnancy-associated glycoprotein (PAG) in sheep with a heterologous (anti-caPAG _55+59_) RIA and its potential for diagnosing pregnancy Theriogenology 2006 66 906 912 10.1016/j.theriogenology.2006.02.031 16566995

[B44] Gonzalez F Sulon J Batista M Cabrera F Calero F Gracia A Beckers JF Early pregnancy diagnosis in goats by determination of pregnancy-associated glycoprotein concentrations in plasma samples Theriogenology 1999 52 717 725 10.1016/S0093-691X(99)00165-X 10734369

[B45] Patel OV Sulon J Beckers JF Takahashi T Hirako M Sasaki N Domaki I Plasma bovine pregnancy-associated glycoprotein concentrations through gestation in relationship to fetal number in the cow Eur J Endocrin 1997 137 423 428 10.1530/eje.0.1370423 9368512

[B46] Abdalla EB Improving the reproductive performance of Egyptian buffalo cows by changing the management system Anim Reprod Sci 2003 75 1 8 10.1016/S0378-4320(02)00225-7 12535580

[B47] De Rosa G Grasso F Brughieri A Bilancione A Di Francia A Napoletano F Behaviour and milk production of buffalo cows as effect by housing system J Dairy Sci 2009 92 907 912 10.3168/jds.2008-1157 19233783

[B48] Lopez-Gatius F Garbayo JM Santolaria P Yaniz P Ayad A Sousa NM Beckers JF Milk production correlates negatively with plasma levels of pregnancy-associated glycoprotein (PAG) during the early fetal period in high producing dairy cows with live fetuses Domest Anim Endocrin 2007 32 29 42 10.1016/j.domaniend.2005.12.007 16423500

[B49] Vandaele L Verberkmoes S El Amiri B Sulon J Duchateau L Van Soom A Beckers JF de Kruif A Use of homologous radioimmunoassay (RIA) to evaluate the effect of maternal and foetal parameters on pregnancy-associated glycoprotein (PAG) concentrations in sheep Theriogenology 2005 63 1914 1924 10.1016/j.theriogenology.2004.08.009 15823348

[B50] Zoli AP Guilbault LA Delahaut P Ortiz WB Beckers JF Radioimmunoassay of a bovine pregnancy-associated glycoprotein in serum: its application for pregnancy diagnosis Biol Reprod 1992 46 83 92 10.1095/biolreprod46.1.83 1547318

[B51] Hashizume K Analysis of uteroplacental-specific molecules and their functions during implantation and placentation in the bovine J Reprod Dev 2007 53 1 11 10.1262/jrd.18123 17332695

[B52] Mamo S Mehta JP McGettigan P Fair T Spencer TE Bazer FW Lonergan P RNA sequencing reveals novel gene clusters in bovine conceptuses associated with maternal recognition of pregnancy and implantation Biol Reprod 2011 85 1143 1151 10.1095/biolreprod.111.092643 21795669

[B53] Thompson IM Cerri RL Kim IH Ealy AD Hansen PJ Staples CR Thatcher WW Effects of lactation and pregnancy on metabolic and hormonal responses and expression of selected conceptus and endometrial genes of Holstein dairy cattle J Dairy Sci 2012 95 5645 5656 10.3168/jds.2011-5113 22863093

[B54] Hue I Degrelle SA Turenne N Conceptus elongation in cattle: Genes, models and questions Anim Reprod Sci 2012 134 19 28 10.1016/j.anireprosci.2012.08.007 22921267

[B55] Zoli AP Ectors F Beckers JF Ruminants gonads as accessory sources of pregnancy specific protein ? [abstract] 72nd Annual Meeting of the Endocrine Society 1990 Philadelphia, Pennsylvania: JP Lippincot Company 373

[B56] Lowry OH Rosebrough NJ Farr AR Randall RG Protein measurement with the folin phenol reagent J Biol Chem 1951 193 265 275 14907713

[B57] Pearson WR Lipman DJ Improved tools for biological sequences Proc Natl Acad Sci USA 1988 85 2444 2448 10.1073/pnas.85.8.2444 3162770 PMC280013

[B58] Vaitukaitis J Robbins JB Nieschlag E Ross GT A method for producing specific antisera with small doses of immunogen J Clin Endocrinol Metab 1971 33 988 991 10.1210/jcem-33-6-988 5316354

[B59] Greenwood FC Hunter WM Glover JS The preparation of 131-I- labelled human growth hormone of high specific radioactivity Biochem J 1963 89 114 123 14097352 10.1042/bj0890114 PMC1202279

